# Chromosome 17p13 deletion is associated with an aggressive tumor phenotype in clear cell renal cell carcinoma

**DOI:** 10.1186/s12957-020-01902-y

**Published:** 2020-06-13

**Authors:** Till Eichenauer, Navid Shadanpour, Martina Kluth, Cosima Göbel, Sören Weidemann, Christoph Fraune, Franziska Büscheck, Claudia Hube-Magg, Christina Möller-Koop, Roland Dahlem, Margit Fisch, Michael Rink, Silke Riechardt, Eike Burandt, Christian Bernreuther, Sarah Minner, Ronald Simon, Guido Sauter, Waldemar Wilczak, Till Clauditz

**Affiliations:** 1 Institute of Pathology, University Medical Center Hamburg-Eppendorf, Martinistr. 52, 20246 Hamburg, Germany; 2 Department of Urology, University Medical Center Hamburg-Eppendorf, Hamburg, Germany

**Keywords:** Renal cell cancer, 17p13 deletion, Fluorescence in situ hybridization, Tissue microarray, Prognosis

## Abstract

**Background:**

Deletions of 17p13 recurrently occur in renal cell carcinoma (RCC) but their prognostic role seems to be uncertain.

**Methods:**

To determine prevalence, relationship with tumor phenotype, and patient prognosis, a tissue microarray containing samples from 1809 RCCs was evaluated using dual labeling fluorescence in situ hybridization (FISH) with 17p13 and chromosome 17 centromere probes.

**Results:**

A 17p13 deletion was found in 72 of 1429 interpretable tumors. The frequency of 17p13 deletions varied greatly between RCC subtypes and was highest in chromophobe RCC (24/72; 33.3%). 17p13 deletions were also found in 35 (3.7%) of 946 clear cell RCC, 9 (4.3%) of 208 papillary RCC, 1 of 121 oncocytomas (0.8%), as well as in several rare cases of comprising 1 of 7 Xp11.2 translocation cancers, 1 of 3 collecting duct carcinomas, and 1 of 20 not otherwise specified (NOS) carcinomas. In clear cell carcinomas, 17p13 deletions revealed a strong and consistent association with higher Fuhrman, ISUP, and Thoenes grade (*p* < 0.0001 each), and linked to advanced tumor stage (*p* = 0.0168), large tumor diameter (*p* = 0.0004), distant metastases (*p* = 0.0077), cancer-specific survival (*p* = 0.0391), and recurrence-free survival (*p* = 0.0072). In multivariate analysis, 17p13 deletions showed in clear cell RCC a dependent prognostic role for established clinical-pathological parameters.

**Conclusion:**

17p13 deletions have a dual role in RCC. They are associated with disease progression in clear cell RCC and possibly other subtypes and they are linked to the development of chromophobe RCC—a subtype with a particularly favorable prognosis.

## Introduction

Renal cell carcinoma (RCC) is the 9th most common tumor worldwide [1]. Its incidence is rising and is highest in countries with high socio-economic status [2]. The reasons behind the growing incidence, especially in developing countries, are a persistently increasing impact of risk factors like smoking, obesity, hypertension, and increasing patient age, but also a more frequent use of common medical imaging types like ultrasound, computer-tomography, and magnetic resonance imaging [3] which lead to an earlier diagnosis. The latter is a likely reason for a reduction of mortality in many countries over the past decade [2].

Localized RCC treatment is generally achieved through radical or partial nephrectomy. Even in metastatic disease, a surgical approach plays a major role if metastases are resectable. Renal cell carcinomas are often resistant to radiation and to most chemotherapies [4]. However, several new drugs such as sunitinib or immune-checkpoint inhibitors have recently yielded better results [5, 6]. Currently, it is thus being evaluated in clinical trials, whether an adjuvant application of these new drugs can improve the prognosis of patients at high risk for disease recurrence or progression after nephrectomy (Keynote-564, iMmotion010, Checkmate-914). Sunitinib was approved by the FDA for this purpose in November 2017. If adjuvant treatment becomes a standard of care, risk stratification will become more important than ever before, to find out which patient is at risk and might benefit from adjuvant therapies. The increasing knowledge about the cell biology of RCC might eventually lead to the identification of molecular tumor features that might help to improve risk stratification.

Chromosomal deletions are often found in many cancers including kidney cancer [7]. In many tumor types, chromosomal deletions were found to be highly prognostic [8–12]. Deletion analysis appears to be particularly well suited for clinical applications and as they are easily detectable by fluorescence in situ hybridization (FISH) resulting in a reproducible yes/no answer (deletion present or not present). The short arm of chromosome 17 (17p) has also been described to be recurrently deleted in kidney cancers, mainly in the chromophobe subtype [13–16]. The prognostic role of 17p13 deletions in RCC is currently unknown, however.

To learn more about the diagnostic and prognostic role of 17p13 deletions in RCC, we thus evaluated more than 1800 kidney tumors—many of which with attached follow-up data—in a tissue microarray (TMA) format by FISH.

## Materials and methods

### Patients

The kidney tumor TMA utilized in this study included samples collected from 1809 patients who underwent surgery at the Institute of Pathology of the University Medical Center Hamburg-Eppendorf (UKE), Germany, between 1994 and 2016. All tumors have been histologically reviewed by two pathologists expertized in genitourinary pathology (FB, CF) at the Institute of Pathology (UKE) following WHO 2016 classification [17]. The International Society of Urological Pathology (ISUP) grading has been applied to each tumor. The TMA consists of four blocks, including one that was built earlier [18]. The TMA construction has been previously described [19]. Supplementary Table 1 summarizes the clinical and pathological parameters of the evaluated cancers. In this report, the mean follow-up period was 48 months. Local laws (HmbKHG, §12,1) and the local ethics committee (Ethics Commission Hamburg, WF-049/09) approved the use of archived diagnostic left-over tissues for the analysis of TMA construction for research purposes as well as the analysis of patient data. All work has been carried out in accordance with the Helsinki Declaration.

### Fluorescence in situ hybridization (FISH)

The general FISH protocol was carried out as described before [20]. The probe set included a spectrum-green labeled 17p13 (targeting the *TP53* gene locus) probe (BACs RP11-89D11, RP11-404G1; Source Bioscience, Nottingham, UK), and a commercial spectrum-orange-labeled centromere 17 reference probe (#06J36-017; Abbott, Chicago, USA).In our evaluation, we excluded tissue spots (tumor or normal cells) without green 17q13 signals or any normal cells as an internal control for successful FISH probe hybridization. For each tissue spot, the predominant FISH signal numbers were recorded. Lack of green signal in ≥ 60% of tumor nuclei indicated homozygous 17q13 deletion, whereas a reduced number of 17p13 probe signals compared to the centromeric 17 probe in ≥ 60% of tumor nuclei indicated heterozygous 17q13 deletion. Thresholds were selected on the basis of the previous study on PTEN deletion results obtained by FISH and single-nucleotide polymorphism (SNP) in a cohort of prostate cancers [21].

### Statistics

The software JMP 12 (SAS Institute Inc., NC, USA) was used for statistical calculations. Contingency tables and the Chi-square test were used to study associations between 17p13 deletions and tumor phenotype. Survival curves were generated using the Kaplan-Meier method and significant survival differences between groups were estimated using the log-rank test. Cox proportional hazards regression analysis was carried out to verify the differences in data for significant associations between pT, ISUP grade, and 17p13 deletions.

## Results

### Technical issues

In total, 1429 out of 1809 (79%) tissue spots provided comprehensive data. Reasons for non-informative cases (380 spots; 21%) included insufficient hybridization with absence of clear 17p13 and/or centromere 17 signals, missing tissue spots, or unclear presence of a cancer tissue on the TMA spot.

### 17p13 deletion in renal cell cancer

**Fig. 1:**
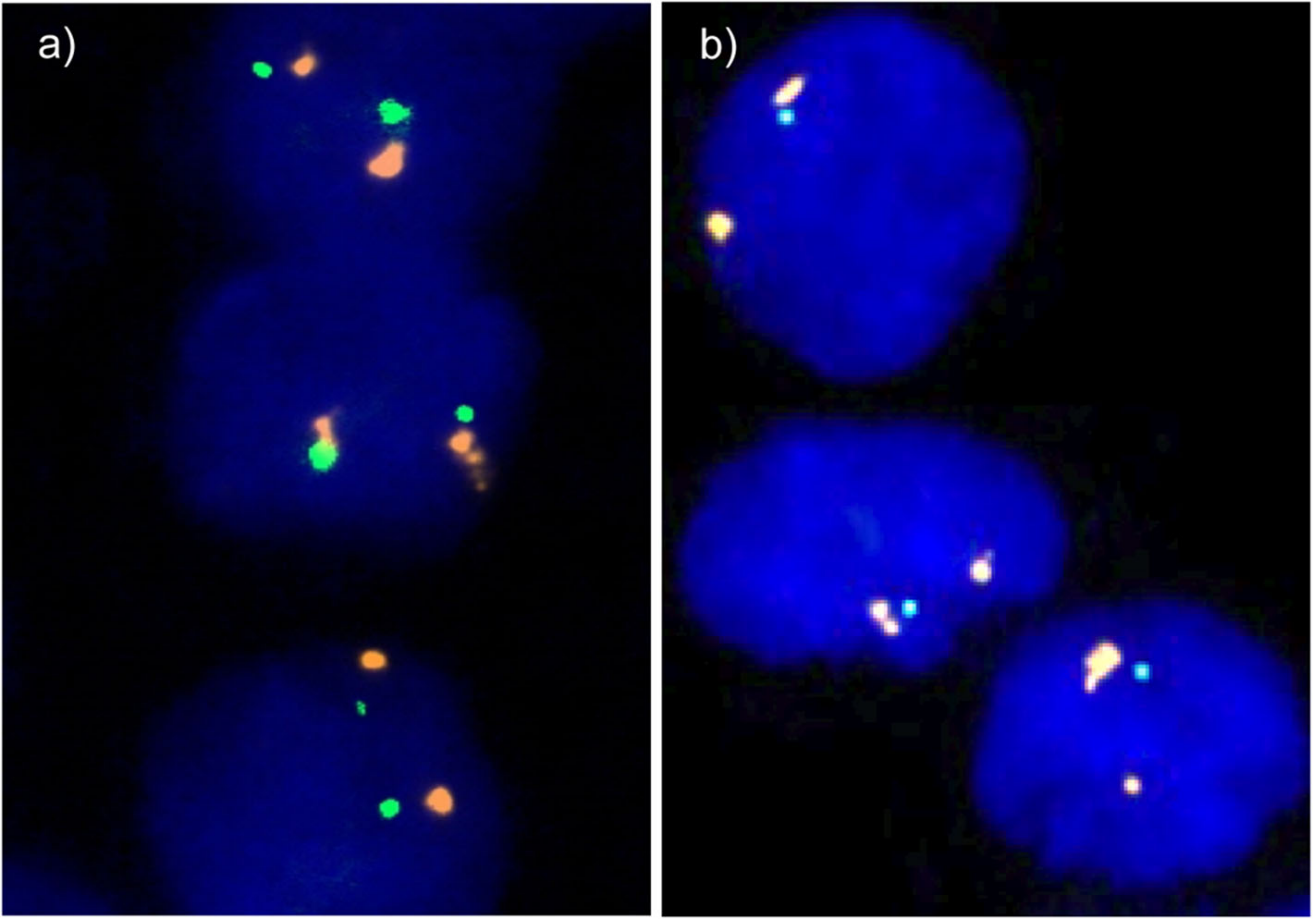
Representative images of FISH analysis. **a** Normal 17p13 copy numbers as indicated by two green 17p13 signals and two orange centromeres 17 signals and **b** heterozygous deletion as indicated by the lack of one green 17p13 signal

Representative images of cancers with and without 17p13 deletion are shown in Fig. 1. A total of 72 out of 1429 analyzable tumor samples (5%) featured 17p13 deletions. The frequency of 17p13 deletions was markedly higher in chromophobe carcinomas (24/72, 33.3%) as compared to clear cell RCC (35/946, 3.7%) and papillary RCC (9/208, 4.3%). 17p13 deletion was present in only one oncocytoma (1/121, 0.8%) and was not seen in 24 clear cell tubulo-papillary RCCs (Table 1). 17p13 deletion was also found in rare subtypes such as in collecting duct carcinomas (1/3, 33%), Xp11.2 translocation RCC (1/7, 14%), and in not otherwise specified tumors (1/20, 5%) (Table 1). In clear cell RCC, 17p13 deletions were strongly linked to ISUP, Fuhrman, and Thoenes grade (*p* < 0.0001 each); pT stage (*p* = 0.0168); and presence of distant metastases (M stage, *p* = 0.0077; Table 2). Clear cell RCC with 17p13 deletions were significantly larger than those without deletions (*p* = 0.0004, Table 3). In papillary and chromophobe RCC, 17p13 deletions were unrelated to tumor phenotype (data not shown) and tumor diameter (Table 3).

**Table 1:** Prevalence of 17p13 deletions in different histological subtypes of renal cell tumors

Renal cell tumor type	Analyzable (***n***)	17p deletion (%)
Clear cell renal cell carcinoma	976	3.7
Papillary renal cell carcinoma	208	4.3
Oncocytoma	121	0.8
Chromophobe renal cell carcinoma	72	33
Clear cell (tubulo) papillary renal cell carcinoma	24	0.0
Carcinoma NOS	20	5.0
Nephroblastoma	14	0.0
Xp11.2 translocation renal cell carcinoma	7	14
Collecting duct carcinoma	3	33
Multilocular cystic clear cell renal cell neoplasm of low malignancy	2	0.0
Metanephric adenoma	2	0.0
Tubulocystic renal cell carcinoma	2	0.0
Mucinous tubular and spindle cell carcinoma	2	0.0
Acquired cystic disease-associated renal cell carcinoma	1	0.0
Cystic nephroma/mixed epithelial stroma tumor	1	0.0
Medullary carcinoma	1	0.0
Neuroendocrine carcinoma	1	0.0
Reninoma	1	0.0

**Table 2:** Associations between 17p13 deletions and pathological parameters of clear cell renal cell carcinomas

	Clear cell carcinomas		
	Analyzable (***n***)	17p deletion	***p*** value
**All**	976	3.7	
**UICC**			
I	420	1.9	0.0524
II	74	5.4	
III	116	4.3	
IV	97	7.2	
**ISUP**			
1	257	1.5	< 0.0001
2	316	1.9	
3	305	5.3	
4	60	15.0	
**Fuhrman**			
1	47	0.0	< 0.0001
2	522	1.5	
3	307	4.9	
4	69	17.0	
**Thoenes**			
1	323	1.2	<0.0001
2	506	3.2	
3	116	13.0	
**Tumor stage**			
pT1	568	2.3	0.0168
pT2	118	5.1	
pT3-4	255	6.3	
**Lymph node metastasis**			
pN0	132	2.3	0.6416
pN1	8	0.0	
pN2	19	5.3	
**Distant metastasis**			
pM0	125	0.8	0.0077
pM1	95	7.4

**Table 3:** Role of 17p13 deletions for tumor size in clear cell renal cell carcinomas

	17p status	***n***	Tumor size (cm)	
			Mean ± sd	*p* value
**All tumors**	Normal	1333	5.1 ± 0.1	0.0046
	Deletion	71	6.2 ± 0.4	
**Clear cell RCC**	Normal	898	5.3 ± 0.1	0.0004
	Deletion	34	7.1 ± 0.5	
**Papillary RCC**	Normal	194	5.1 ± 0.3	0.4142
	Deletion	9	6.1 ± 1.2	
**Chromophobe RCC**	Normal	47	5.0 ± 0.4	0.8695
	Deletion	24	5.1 ± 0.6	

### Associations with patient survival

**Fig. 2:**
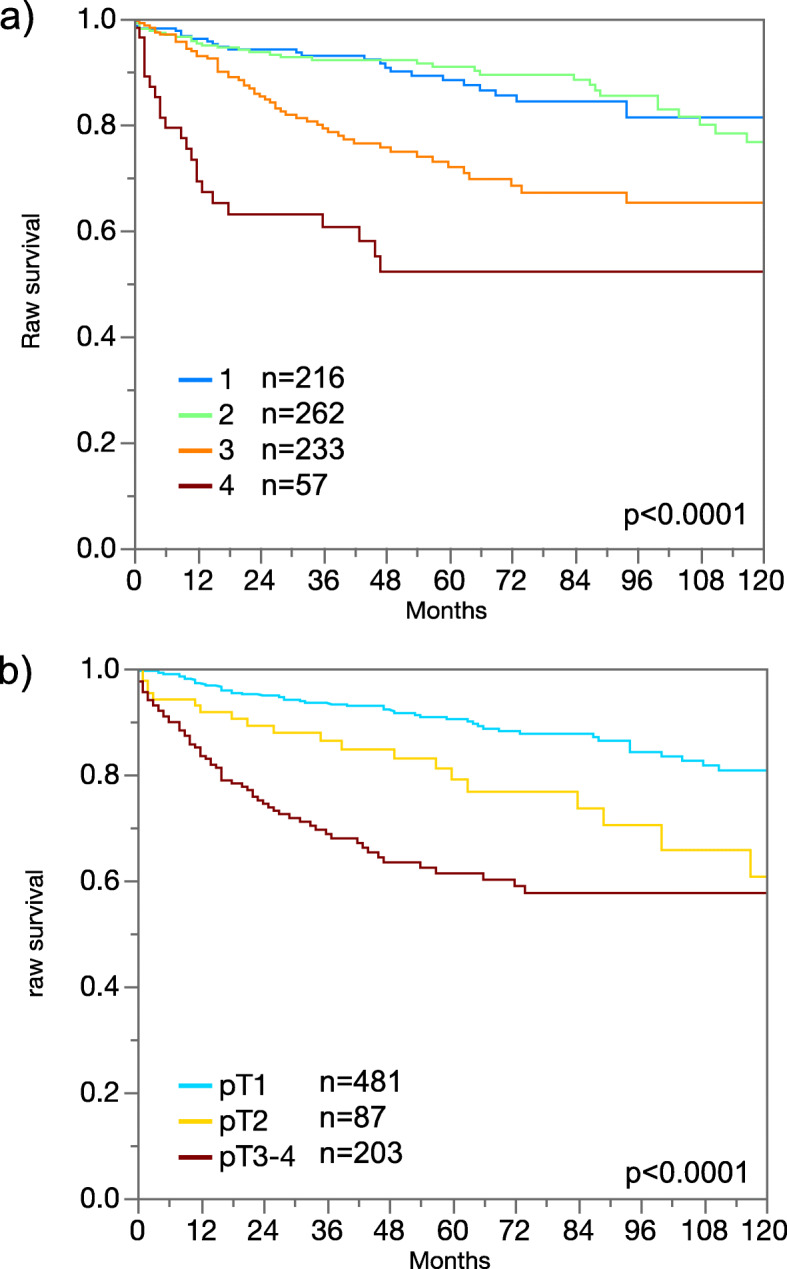
Prognostic relevance of **a** ISUP grade and **b** tumor stage in clear cell renal cell carcinomas

**Fig. 3:**
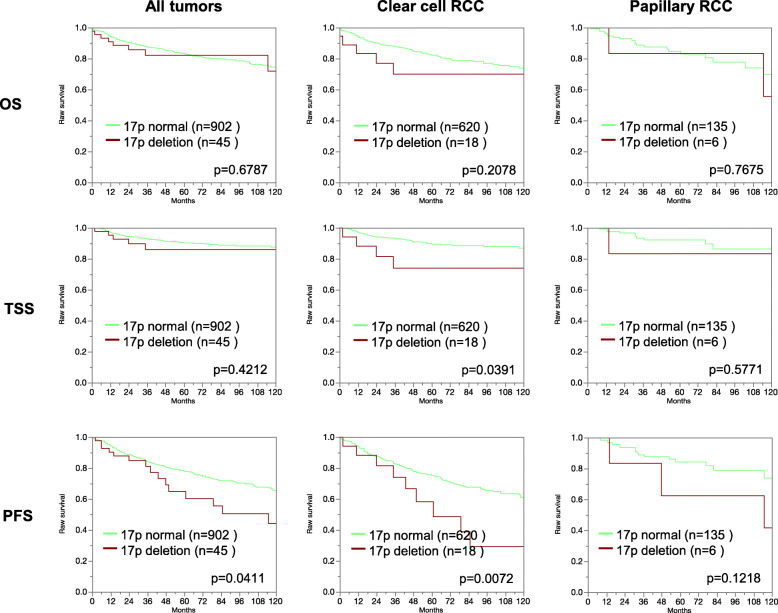
Association between 17p13 deletion and overall survival (OS), tumor-specific survival (TSS), and progression-free survival (PFS) in all tumors as well in the subsets of 894 clear cell renal cell carcinoma and 197 papillary renal cell carcinomas

In the present study, follow-up data for 789 clear cell and 177 papillary cancers were accessible. Figure 2a, b shows associations between ISUP grade and tumor stage with the survival data for our clear cell cancers. These findings demonstrate the validity of our follow-up data. 17p13 deletions were significantly associated with progression-free survival in the joint analysis of all tumors (*p* = 0.0411). Also, in the largest subgroup of clear cell cancers, 17p13 deletions were significantly associated with shortened cancer-specific (*p* = 0.0391) and recurrence-free (*p* = 0.0072) survival. 17p13 deletions were unrelated to patient prognosis in papillary cancers, probably due to the small minority of cases with 17p13 deletion. All data are shown in Fig. 3. Follow-up data were insufficient (only 73 patients with follow-up information) to analyze the prognostic role of 17p13 deletions in chromophobe carcinomas. In a multivariate analysis including pT, pN, M status, and ISUP grade, 17p13 deletions did not show an independent prognostic significance for all endpoints (supplement table 2).

## Discussion

The results of this study identify a dual role of 17p13 deletions in RCC. These deletions are involved in the progression of clear cell and possibly other RCCs. They are also relevant for the development of chromophobe RCC, a less aggressive kidney cancer subtype.

The fraction of 17p13-deleted clear cell RCCs was 5% in our FISH analysis. A lower percentage than in most earlier studies, which described 17p deletions in 7–11% by classical comparative genomic hybridization (CGH) [22, 23], 4–77% by loss of heterozygosity (LOH) analysis [24–40], 20% by restriction fragment length polymorphism (RFLP) analysis [30, 41], 31–53% by single-nucleotide polymorphism (SNP) array hybridization [42, 43], 12% by classical cytogenetics [44], and 18% using FISH [45]. Next-generation sequencing data available from The Cancer Genome Atlas (TCGA) [7] show 17p13 (*TP53*) deletions in 8% of 345 analyzed clear cell RCC. It appears obvious that these differences in the frequency of reported 17p deletions are at least partly connected to technical issues related to the different methods. LOH, CGH, RFNP, SNP, and NGS share the disadvantage that the analysis is performed on isolated DNA, which always bears the risk of DNA contamination from adjacent non-neoplastic cells such as stroma, immune cells, blood vessels, and so on. In addition, most studies used less sensitive methods than this study. FISH is considered the gold standard for detection of deletions. FISH allows a precise cell by cell assessment of the copy number of genomic regions of interest. FISH is independent of the presence and quantity of inflammatory or stroma cells. Some “false deletions” can be assumed in FISH analyses because some signals are always missing due to truncated cell nuclei that are incompletely represented on a tissue slide measuring only 3–4 μm in thickness. A rigorous cut-off of 60% of tumor cells having less 17p13 than centromere 17 signals was thus selected in this project to define 17p13-deleted tumors. This is based on the assumption that clinically relevant intratumoral heterogeneity will not occur in a TMA spot measuring 0.6 mm in diameter. In an earlier study, we had found a 100% concordance between FISH and array CGH data for identifying PTEN deletions using this definition for deletion [21].

17p13 deletions were clearly associated with an unfavorable tumor phenotype and poor prognosis in clear cell RCC. Given the striking association of 17p13 deletions with tumor grade and stage, it is possible that the rather low number of 17p13-deleted cases in our study is due to the consecutive nature of our cohort including a large number of pT1 tumors. Considering the low frequency of 17p13 deletions and the low number of papillary carcinomas in this study, it is not surprising that clear-cut associations between 17p13 deletions and unfavorable tumor features could not be found in this RCC subtype. The significant link between 17p13 deletions and progression-free survival would be consistent, however, with 17p13 deletions representing a universal feature of disease progression in RCCs derived from the proximal tubule. The tumor suppressor p53 residing on chromosome 17p13 is an apparent candidate target gene of 17p13 deletions. An altered p53 function occurs in less than 5% of clear cell and papillary RCCs [46]. Data from The Cancer Genome Atlas (TCGA) PanCancer database show concomitant p53 mutations only in 4 of 27 clear cell RCCs with 17p13 deletions [7]. It is possible, however, that a reduced p53 function in 17p13-deleted cells contributes to increased potential for further progression.

A large number of candidate prognostic markers have been suggested for kidney cancer in the past. These include deletions of chromosomal material such as losses of 1p, 3p, 8p, 9p, and 14q in clear cell cancers [47–52], 3p, and X-chromosome loss in papillary cancers [53, 54], and monosomies of chromosome 1, 2, 10, 13, 17, and 21 in chromophobe RCC [55]. However, none of these markers was so far strong enough to compete with classical histo-pathological prognostic parameters in multivariable analyses. As a consequence, no molecular marker has entered routine clinical diagnostic procedures so far. Nevertheless, these abovementioned findings suggest that genomic instability is typically related with adverse renal cell cancer phenotype. It is, therefore, well possible that combinations of multiple genomic deletions may better predict the clinical course than one of these deletions alone. This is supported by studies showing that combinations of multiple deletions such as 3p and 14q [47] or ploidy changes [56] are particularly strongly linked to poor prognosis. Future molecular prognostic test may, therefore, combine multiple genomic alterations. Although the 17p deletion did not show independent prognostic value in this study, it may be a valuable marker to be included in such potential future multiparametric tests, particularly for clear cell RCC.

That 17p13 deletions were strongly linked to chromophobe tumor subtype in this study fits well with data from earlier studies. As seen for clear cell RCCs, other investigators had earlier described even higher frequencies of 17p13 deletions in chromophobe cancers than the 33% in our study. Speicher et al. found a chromosome 17 loss in 13/17 cases of chromophobe RCC using CGH [13]. Yusenko et al. found 17p13 deletions in 90% of 30 chromophobe RCC using SNP array analysis [15]. Nagy et al. described LOH in 90% of 21 chromophobe RCC by microsatellite allelotyping [16]. Brunelli et al. found 17p13 deletions in 9 of 11 chromophobe cancers by FISH [14]. The TCGA database identified 17p13 deletions in 75% of 65 analyzed chromophobe RCCs [7]. The clinical outcome of chromophobe RCC is generally better than seen in clear cell RCC. It is thus unlikely, that the particular role of 17p13 deletions in these tumors is related to the *TP53* gene, inactivation of which is generally associated with aggressive cancer [46].

Increasing evidence suggests that the association of 17p13 deletions with chromophobe RCC subtype may be driven by the Folliculin (FLCN) gene on 17p11.2. FLCN interacts with TFE3 and the Wnt pathway and plays a critical role for the exit from human pluripotency [57]. FLCN germline mutations cause the Birt-Hogg-Dubé (BHD) syndrome characterized by benign hair follicle hamartomas, spontaneous pneumothorax, lung cysts, and an increased risk for renal carcinoma. Patients with BHD syndrome tend to develop RCC, which primarily are chromophobe RCC (34%) or renal hybrid oncocytic/chromophobe tumors (50%) with areas reminiscent of chromophobe RCC and oncocytoma [58]. According to the cBioPortal database [7], FLCN mutations were not found in 65 chromophobe carcinomas that had been sequenced in the TCGA project [7]. This suggests that the rare BHD syndrome is only linked to a small fraction of chromophobe RCCs. Based on all these data, it is tempting to speculate, that partial inactivation of FLCN in 17p13-deleted cells from the distal tubule favors the development of chromophobe cancers. It is of note that 17p13 deletions were exceptionally rare (0.8% deleted cancers) in oncocytomas. Oncocytomas, the benign counterpart of chromophobe carcinoma, share the origin from the distal tubule with chromophobe carcinoma but are very uncommon in BHD syndrome. Given the tight link of 17p13 deletion and chromophobe RCC, one might assume that an additional loss of 17p13 occurring in an oncocytoma could result in a progression to renal hybrid oncocytic/chromophobe tumors, the most common RCC in BHD syndrome.

## Conclusion

In summary, our data provide evidence for a dual role of 17p13 deletions in RCC. In cells from the distal tubule, 17p13 deletions contribute to the development of chromophobe RCC. In clear cell and papillary RCC derived from the proximal tubule, they are linked to disease progression. Together with other molecular parameters, the assessment of 17p13 deletions may have clinical utility for prognosis assessment in clear cell RCC and perhaps also papillary RCC.

## Supplementary information

**Additional file 1: Table S1.** Pathological and clinical data of the arrayed renal cell tumors.

**Additional file 2: Table S2.** Multivariate analysis in clear cell renal cell cancers using the endpoints overall survival (OS), tumor specific survival (TSS) and progression-free survival (TFS).

## Data Availability

Data will be made available upon reasonable request.

## Abbreviations

RCC: Renal cell carcinoma
FISH: Fluorescence in situ hybridization
NOS: Not otherwise specific
FDA: Food and Drug Administration
TMA: Tissue microarray
UKE: University Medical Center Hamburg-Eppendorf
WHO: World Health Organization
ISUP: International Society of Urological Pathology
PTEN: Phosphatase and tensin homolog
SNP: Single-nucleotide polymorphism
CGH: Comparative genomic hybridization
LOH: Loss of heterozygosity
RFLP: Restriction fragment length polymorphism
TCGA: The Cancer Genome Atlas
BHD: Birt-Hogg-Dubé
